# Severe fetal intracranial hemorrhage: Congenital Cytomegalovirus infection may play a role? A case report and review of literature

**DOI:** 10.1016/j.idcr.2021.e01188

**Published:** 2021-06-11

**Authors:** Letizia Capasso, Clara Coppola, Maria Vendemmia, Serena Salomè, Valentina Esposito, Chiara Colinet, Carolina Porfito, Francesco Raimondi

**Affiliations:** Neonatology and NICU, Department of Translational Medical Sciences, University Federico II, Naples, Italy

**Keywords:** CMV infection, Intracranial fetal hemorrhage, Neonate, Prenatal diagnosis, Cytomegalovirus

## Abstract

Cytomegalovirus (CMV) is the most common cause of congenital infection, with a wide spectrum of clinical manifestations and different grade of severity. We report the case of a male baby born at term with an early prenatal diagnosis of severe intracranial hemorrhage (ICH), with no other evident risk factors. Urine and blood sample were tested for CMV-DNA, and diagnosis of congenital CMV infection was established. This case describes intracranial hemorrhage as uncommon although possible sign of early fetal CMV infection. Considering that pathogenic factors cannot be defined in 25 % of term neonates with ICH, this case report highlights the importance of CMV screening in pregnant women and in term infants with prenatal ICH of unknown origin.

## Highlights

• 25 % of severe intracranial hemorrhage (ICH) in at term neonates have unknown origin.
• In case of ICH in at term neonate CMV fetal infection needs to be excluded.

## Introduction

CMV is the most common cause of congenital infection with a wide spectrum of manifestations. Approximately 10 % of neonates with congenital CMV (cCMV) infection are symptomatic at birth with clinical manifestations including jaundice, petechiae, purpura, hepatosplenomegaly, microcephaly and neurological symptoms, including intracerebral calcifications (typically periventricular), sensorineural hearing loss, cerebellar and hippocampal hypoplasia, cortical dysplasia (such as pachygyria, micropolygyria and lissencephaly) and retinitis. Neurodevelopmental impairment in childhood is common [[1], [2], [3]]. Intraventricular hemorrhage is a rare complication of cCMV infection and has been reported either in very premature infants or in association with thrombocytopenia [4,5]. We report a rare case of fetal ICH diagnosed at birth as cCMV infection without thrombocytopenia. The diagnosis of cCMV infection in neonates include real-time PCR of saliva, urine or both, as soon as possible after birth within the first 3 weeks of life. Valganciclovir treatment for 6 months in congenitally infected neonates with moderately to severely symptomatic disease could improve audiological and neurodevelopmental outcomes at 2 years of age [6,7]. Early diagnosis of cCMV is mandatory to start therapy within the first month of life.

## Case presentation

MR was born at 38 weeks of gestational age (GA) from elective cesarean section. At birth: weight 2350 g (5−10° pt), length 45 cm (5° pt), head circumference 31 cm (<5° pc). Doppler flow at obstetric ultrasounds was regular. Placenta normal in shape and perfusion. The Apgar score was 8 at 1st min., 9 at 5th min.

At beginning of pregnancy, the mother was diagnosed as immune to rubella and she was negative for toxoplasmosis specific antibodies (IgG and IgM) throughout all the pregnancy. Only one determination of serological status for CMV at 11th week of GA was available(IgG positive and IgM negative). A maternal febrile episode in peri-conceptive period was reported. No maternal trauma reported in early pregnancy. Parents were not consanguineous and there was no family history of any bleeding disorders or stroke. No maternal use of cocaine or warfarin or anticonvulsant was reported.

**Fig. 1:**
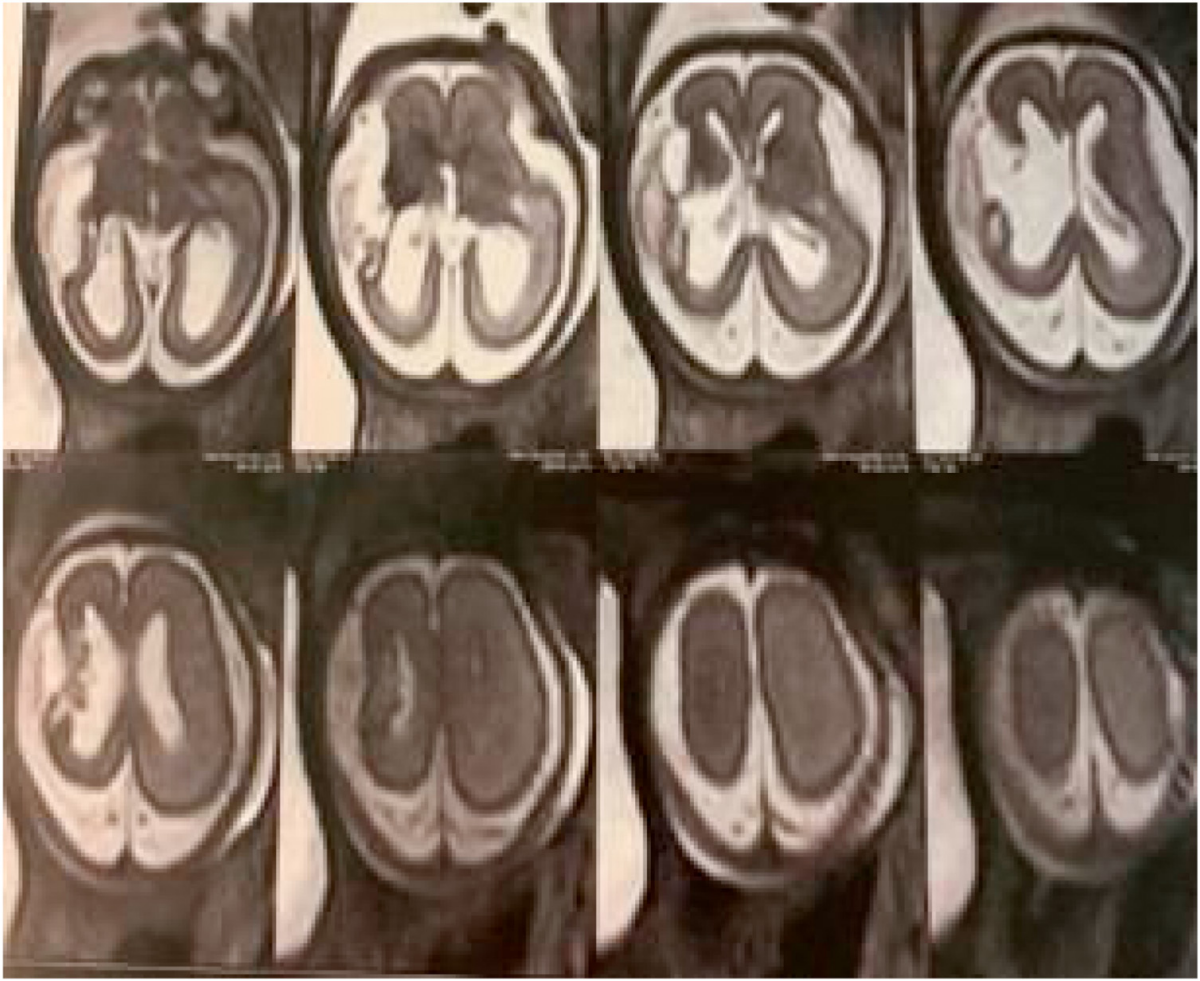
Fetal cerebral MRI. Fetal MRI scan (25 weeks of gestation) showing extensive poroencephaly (2.3 cm of diameter) in the fronto- temporal and insular zone with “ex vacuo” enlargement of the right lateral ventricle, reduced volume of the right cerebral hemisphere. An anomalous development of the middle-lower right frontal cortex is highlighted, which shows a polylobulated aspect due to reduced amplitude of the furrows and increased number of turns (suspect of micropolygyria) extended up to the right parietal lobe.

A prenatal diagnosis of ICH was suspected during an ultrasound performed at 20th week of GA and it was confirmed at 25th week of GA through a fetal brain Magnetic Resonance Imaging (MRI) scan (Fig. 1).The prenatal MRI showed a porencephaly in the right fronto- temporal and insular zone with dilatation of the right lateral ventricle, reduced volume of the right cerebral hemisphere. In addition, it showed anomalous development of the right frontal cortex with a polylobulated aspect due to reduced amplitude of the furrows and increased number of turns (suspect of micropolygyria). No arterial nor venous abnormalities were reported.

Because of these prenatal findings, immediately after birth the neonate was admitted in our Neonatal Intensive Care Unit for further evaluations.

At birth physical examination revealed micro-purpuric elements on face and upper thorax, microcephaly, mild upper limbs muscular hypertonicity, abnormal deep tendon reflexes (plantar cutaneous reflex in extension bilaterally, absent left knee jerk), jaundice.

Laboratory evaluations were performed, including complete blood cell count, coagulative profile and liver and kidney function tests. Examinations for suspected congenital coagulopathy causing cerebral bleeding were performed (i.e. protein C and S, dosage of coagulative factors II, V, VII, X, XI, XIII, von Willebrand, IX, alfa2 antiplasmin, APC resistance, lupus anticoagulant and anti-cardiolipin/beta 2 glycoprotein antibodies): all these exams resulted normal. Moreover, gene sequencing for MTHFR/Leiden Factor polymorphism resulted wild type. Screening for metabolic diseases at birth was negative.

**Fig. 2:**
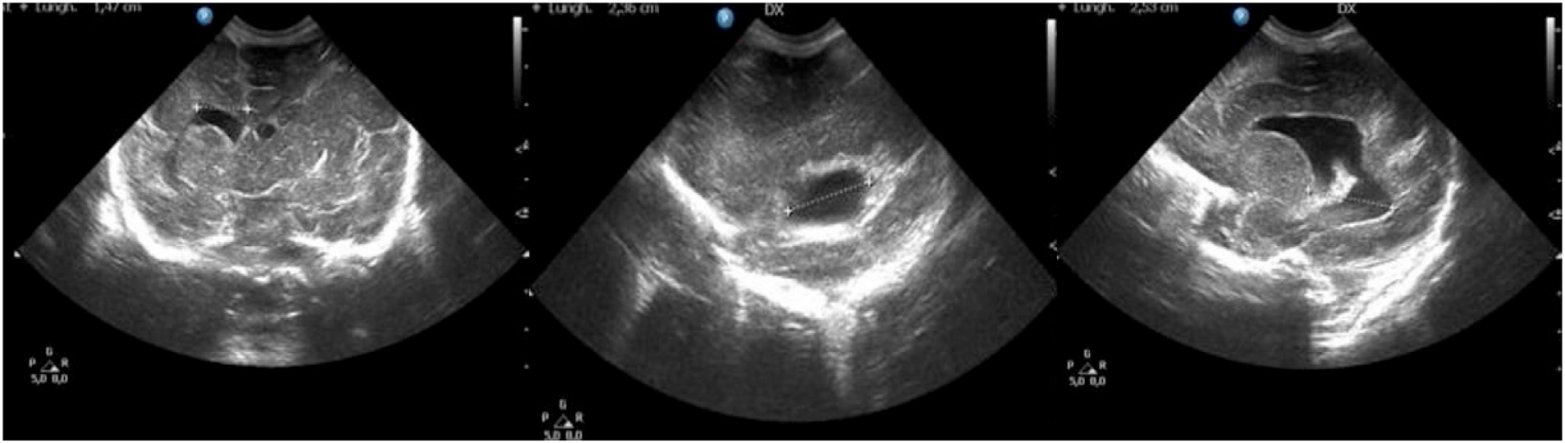
Cerebral Ultrasound performed after birth. Cerebral ultrasound performed on the first day of life both in coronal and sagittal images. It shows left cerebral hemisphere prevailing on the right, a poroencephalic area of 2.3 cm of diameter in the right side, with “ex vacuo” ventricle enlargement (Ventricular Index1.4 cm. Occipital Thalamus Distance 2.5 cm) till Silvian fissure.

Cranial ultrasound performed on first day of life showed left cerebral hemisphere prevailing on the right, a poroencephaly (2.3 cm of diameter) on the right side with “ex vacuo” ventricle enlargement (Ventricular Index1.4 cm, Occipital Thalamus Distance 2.5 cm) till Silvian fissure. A germinolytic cyst on the left side and hyperechoic spots in the left peritrigonal parenchyma were found. Corpus callosum was poorly visible in its anterior portion. Third and fourth ventricles were normal-sized (Fig. 2).

**Fig. 3:**
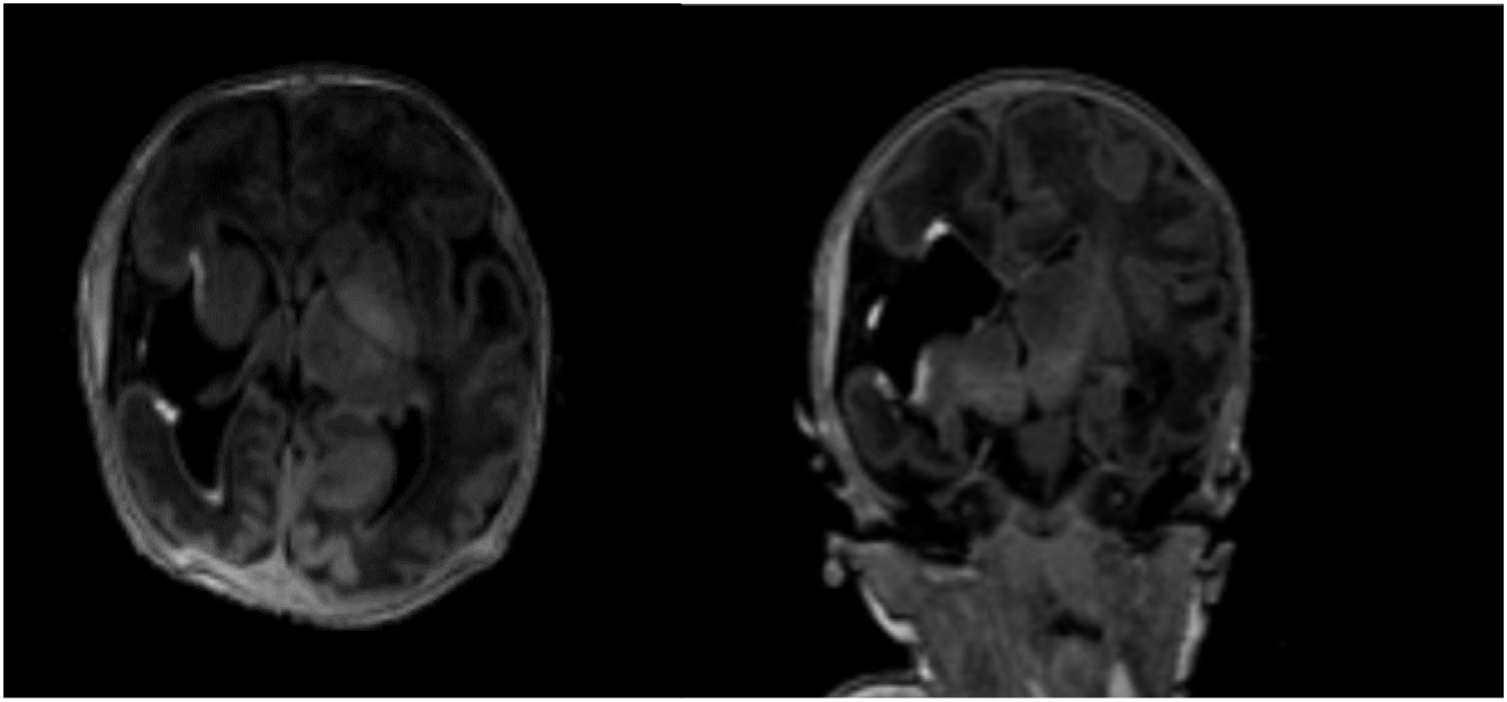
Cerebral MRI performed after birth. Axial and coronal MRI T1 sequences showing extensive poroencephalic area in the right front-temporal and insular zone with “ex vacuo” enlargement of the right lateral ventricle, reduced volume of the right cerebral hemisphere. Abnormal development of the middle-lower right frontal cortex with a polylobate appearance due to reduced furrows width and increased number of turns confirming the micropolygyria extended up to the right parietal lobe.

A postnatal MRI scan confirmed the previous finding. It showed poroencephaly in the right fronto-temporal and insular zone with “ex vacuo” ventricle enlargement, reduced volume of the right cerebral hemisphere, with striatum nucleus and thalamus partially involvement with wallerian degeneration of the corticospinal bundle. MR angiography did not show arterial and venous malformations nor thrombosis. An abnormal development of the middle-lower right frontal cortex was highlighted, which showed a polylobate appearance due to reduced furrows width and increased number of turns confirming the micropolygyria extended up to the right parietal lobe (Fig. 3).

The electroencephalogram showed cortical abnormal activity in frontal-temporal-parietal cortex.

Abdomen and cardiac ultrasound examinations, audiological (TEOAE) and ophthalmological evaluation were performed and all were normal.

Considering the micropolygyria at MRI scan, on the fifth day of life we searched for CMV DNA in body fluid and we detected in urine 10.273.900 copies/mm^3^ and 449 copies/ mm^3^ in blood. Therefore, the patient received diagnosis of congenital CMV infection and it was classified as a severe symptomatic one because of central nervous system involvement (microcephaly, micropolygyria and a wide malacic area in right half brain as consequence of ICH, with “ex vacuo lateral ventricle enlargement”). According to current guidelines on the 15th day of life he started antiviral treatment with oral valganciclovir (16 mg/kg/dose, administered orally twice daily) for 6 months.

Discharged at the 17th day of life, the patient was admitted in our outpatient program for perinatal infectious diseases. No side effects were reported as consequence of the antiviral therapy. Evoked auditory brainstem response and ophthalmological evaluations were performed and resulted normal. Neurological examination at three months of age underlined persistence of neurological impairment more evident at the left side and he was admitted to physio-kinesiotherapy and neuro developmental follow up. At 18 month of life he presented global delay in development with pyramidal signs in the left hemisome. In particular he achieved head and trunk control and from the supine position he reached the prone and vice versa; with double support he maintained the standing position and took a few steps. His language were characterized by lallation and some simple bisyllabic phonemes. The EEG trace showed anomalies with potential epileptiform significance on the right hemisphere without critical episodes so that he did not start antiepileptic therapy.

## Discussion

Intracranial hemorrhage is a rare complication of cCMV infection. Two different hypotheses have been advanced to explain this condition.

One hypothesis considers CMV as cause of direct neuronal damage, due to its neurotropic nature, especially in early gestational age, resulting in cortical dysplasia and hippocampus and cerebellum hypoplasia.

A second hypothesis considers CMV as cause of vasculitis that can affect blood vessels of the central nervous system, triggering thrombotic or hemorrhagic processes, even in absence of apparent coagulopathy [8,9]. This type of complication has been reported mostly in extremely premature infants [10] and less frequently in term neonates. As differential diagnosis, fetal stroke has been reported also in association with thrombocytopenia, prothrombotic disorders, bleeding diathesis, metabolic diseases, maternal trauma and use of warfarin, anticonvulsant and cocaine [[11], [12], [13]] that were excluded in our patient. ICH has also been reported in fetuses proven to have COL4A1 mutations but the absence of positive familiar anamnesis for stroke and the normality of eye exam led us to exclude it [13].

Suksumek et al. [14] reported a case of term neonate with congenital CMV infection and intraventricular hemorrhage with a normal platelet count and coagulation profile. It was diagnosed later in pregnancy at 38 weeks of gestation, in a baby without growth retardation.

Our case, apparently very similar to this one, differs however for the earlier gestational age at CMV infection. In fact, the intracranial hemorrhage in utero was suspected at 20th week of GA and the earlier infection is in line with the finding of micropolygyria, microcephaly with global growth retardation and the severe neurological impairment showed since birth. The two cases may share the common origin but the early onset in our case lead to a different and more severe clinical picture. Moreover, in our case there was a very complex cerebral picture not only consequence of an intraventricular hemorrhage. In fact, cerebral fetal damage did not probably originate from the germinal matrix, related for example to the immaturity of its vessels, because it would have been restricted to the germinal matrix or at least extent into the lateral ventricles. We speculated that our patient suffered from a complex ischemic-hemorrhagic phenomenon in utero caused by CMV infection, which covered a wide area of parenchyma, including also but not only germinal matrix, where neural precursors originate. In prenatal MRI, an abnormal signal pattern in sub plate was reported and it may correlate with an anomalous cortical organization, which results in the micropolygyria in the same areas detected in the post-natal MRI.

Maternal history may suggest a very early infection in the first trimester or a reinfection [15] in the early second trimester for the presence of micropolygyria that is expression of an early brain damage associated to cCMV infection.

Parents of neonate received a full prenatal counseling on ICH diagnosis otherwise, they decided to continue pregnancy and asked for the full medical care for the neonate.

In conclusion, in our patient, diagnosis of fetal ICH forerun the diagnosis of congenital CMV. ICH affects 0.5/1000 of symptomatic term infants and in 25 % of these cases, pathogenic factors cannot be defined. This case report highlights the importance of testing pregnant women and neonate for CMV infection in case of unexplained fetal intracranial bleeding as a possible etiology.

## Authors’ contribution

Conceptualization: LC, FR.

Supervision. LC, FR.

Writing and editing: LC, CCoppola, MV, SS.

Data Collection: VE, CColinet, CP.

Review: LC, MV, SS.

## Ethical approval

Not applicable.

## Consent

The parents of the neonate gave as consent for publication of the clinical history and images.

## Funding

No funding to declare.

## Declaration of Competing Interest

The authors declare that the research was conducted in the absence of any commercial or financial relationships that could be construed as a potential conflict of interest.
